# Research on the Integrated Navigation Technology of SINS with Couple Odometers for Land Vehicles

**DOI:** 10.3390/s20020546

**Published:** 2020-01-19

**Authors:** Jiaxin Gao, Kui Li, Jiyang Chen

**Affiliations:** School of Instrumentation and Optoelectronic Engineering, Beihang University, Beijing 100191, China; gaojiax@buaa.edu.cn (J.G.); chenjiyang@buaa.edu.cn (J.C.)

**Keywords:** integrated navigation system, odometer, strap-down inertial navigation system, Kalman filter

## Abstract

Autonomous and accurate acquisition of the position and azimuth of the vehicle is critical to the combat effectiveness of land-fighting vehicles. The integrated navigation system, consisting of a strap-down inertial navigation system (SINS) and odometer (OD), is commonly applied in vehicles. In the SINS/OD integrated system, the odometer is installed around the vehicle’s wheel, while SINS is usually installed on the base of the vehicle. The distance along SINS and OD would cause a velocity difference when the vehicle maneuvers, which may lead to a significant influence on the integration positioning accuracy. Furthermore, SINS navigation errors, especially azimuth error, would diverge over time due to gyro drifts and accelerometer biases. The azimuth error would cause the divergence of dead-reckoning positioning errors with the distance that the vehicle drives. To solve these problems, an integrated positioning and orientation method based on the configuration of SINS and couple odometers was proposed in this paper. The proposed method designed a high precision integrated navigation algorithm, which compensated the lever arm effect to eliminate the velocity difference between SINS and odometers. At the same time, by using the measured information of couple odometers, azimuth reference was calculated and used as an external measurement to suppress SINS azimuth error’s divergence over time, thus could further improve the navigation precision of the integrated system, especially the orientation accuracy. The performance of the proposed method was verified by simulations. The results demonstrated that SINS/2ODs integrated system could achieve a positioning accuracy of 0.01% D (total mileage) and orientation accuracy of ±30″ by using SINS with 0.01°/h Fiber-Optic Gyroscope (FOGs) and 50 µg accelerometers.

## 1. Introduction

In modern warfare, land-fighting vehicles are often threatened by remote detection systems and precision strike systems and usually need to move quickly to increase their survivability [1], which means that the land vehicles must have the ability to obtain the position and azimuth autonomously and accurately and could launch immediately when parking or even traveling. Therefore, it is very important to improve the land vehicles’ maneuverability, viability, and rapid response capability by devising an integrated navigation technique that can obtain the position and azimuth information of vehicles autonomously and accurately [2].

To achieve the above goals, the integrated navigation system of the strap-down inertial navigation system (SINS) and odometer is applied in land vehicle applications since it can autonomously obtain relatively high-accuracy navigation reference information [3,4]. In this way, the land vehicle departs from a known coordinate position to perform a task, and it can obtain position and attitude information immediately at any launch point. Compared with the integrated navigation system of SINS and global navigation satellite system (GNSS), the SINS and odometer integrated navigation system does not need to acquire external signals and is not vulnerable to external interference. So, it has significant advantages in modern warfare [5,6,7].

The research of this integrated navigation system mainly focuses on online calibration and compensation of integrated navigation system error parameters and the measurement fault detection of odometer caused by land vehicle jumping, taxiing, skidding, and sliding. In [8], the odometer aided in-motion alignment method was proposed, and the error of odometer was comprehensively modeled as a part of an integrated dynamic model. The effectiveness of the method was verified by ground alignment experiments. In [9], the SINS and odometer integrated navigation system adopted a Kalman filter to design an alignment method to assist the SINS for initial alignment on the moving base. A high accuracy integrated algorithm was proposed in [10], and in the algorithm, whether the odometer is faulty was determined by the difference of mileage increments of the SINS and OD outputs. In [11], a fault detection method based on the adaptive Kalman filter was introduced to judge the odometer condition. It could effectively improve navigation accuracy and detect the odometer fault in time.

Recently, more and more researchers paid attention to the rotational inertial navigation system INS (RINS) [12,13,14]. A new integrated navigation system of FOG single-axis rotational INS (FRINS) and OD was presented. It adopted the rotation modulation technique to suppress the inertial sensor errors, especially *z*-axis FOG drift; at the same time, it combined the FRINS with odometer to suppress the divergence of INS error over time. The experiments showed that the positioning accuracy of the vehicle was effectively enhanced, and the cost of the system was saved [15].

The literature presumes that the installation positions of SINS and odometer are exactly the same. In a practical situation, however, it is impossible to ensure that the installation positions of SINS and odometer are completely a coincidence. When the land vehicle makes an azimuth or a pitch turn, the longer lever arms and higher turning speed that SINS/ODs have, the more velocity difference it would have. Therefore, the lever arm effect is a crucial factor that affects the positioning and orientation accuracy of the integrated navigation system. In the existing literature, the lever arm effect of the SINS and odometer integrated system is only simply explored.

Moreover, a single odometer only can provide velocity or mileage measurement information, and it is unable to provide azimuth measurement reference information for the integrated navigation system to solve the problem of the azimuth error’s divergence over time. At the beginning of the land vehicle traveling, the errors of the integrated navigation system cannot be estimated precisely and compensated correctly, which results in large navigation error and affects the positioning and orientation accuracy of the land vehicle.

In this paper, a new integrated navigation method, based on FOG SINS and couple odometers, was proposed to improve the navigation precision, especially the orientation precision. In the proposed method, a high-precision integrated navigation algorithm was established, and the algorithm could effectively compensate for the effect of the lever arm and eliminate the difference of measurement information between SINS and odometers. Besides, by using the couple odometers, the azimuth reference information was calculated and used as the external measurements to suppress SINS azimuth errors’ divergence over time. In this case, the proposed method could effectively improve the positioning accuracy, especially the orientation accuracy of land vehicles. The proposed method was implemented and verified by several simulations, and the results demonstrated that compared with the traditional single odometer and SINS integrated navigation method, the proposed method could achieve the positioning accuracy of 0.01% D (total mileage) and orientation accuracy of ±30″, respectively, by using SINS with 0.01°/h FOGs and 50 µg accelerometers.

The paper is organized as follows: the configuration of SINS and couple odometers integrated navigation system is described in Section 2, and then the working process of the proposed method is presented. In Section 3, the velocity and azimuth error models of odometer are deduced step by step. In Section 4, the state equations and measurement equations of the proposed integrated method are established in detail. Simulations, as well as their results, are conducted and analyzed in Section 5. Finally, conclusions are drawn in Section 6.

## 2. Principle of the Proposed Method

### 2.1. System Configuration and Lever Arm Effect

In order to clearly illustrate the configuration of the integrated system proposed in this paper, the coordinate systems involved were configured as follows: The integrated system chose the east-north-up geographic coordinate system as the navigation frame (n-frame, O-ENU). The axis direction of body coordinate system (b-frame, O-XYZ) was defined as: *x*-axis was pointing to the right side of the vehicle, *y*-axis was along the forward side of the vehicle, *z*-axis was pointing to the vertical, and *x*, *y*, and *z* axes were in a right-handed coordinate system.

**Figure 1:**
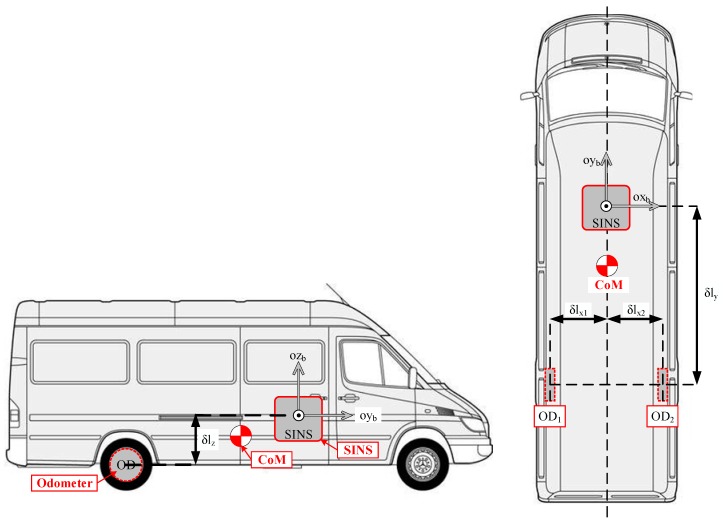
The configuration of the integrated navigation system.

The configuration of the proposed integrated system is briefly illustrated in Figure 1. The system was mainly composed of SINS and a couple of odometers. The SINS was fixed on the front base of the land vehicle, and it contained three accelerometers and three FOGs, which have many advantages, including low cost, small size, lightweight, short start-up time, high reliability, etc. The Y-axis of SINS was along the forward side of the vehicle. The couple optical odometers, as shown on the left of Figure 1, were separately mounted on two rear wheels of the land vehicle, by which the increment data of the land vehicle could be measured in real-time. The photodetector and the grating were the main components of the odometer. 

After the system installation and pre-calibration, it was assumed that the body frame of the SINS coincided with the vehicle’s frame. The relative installation positional relationship between the SINS and odometers is shown in Figure 1, as well as the center of mass (CoM) of the vehicle. The measurement point of the odometer relative to the SINS could be expressed in b-frame as:

(1) $$\delta l^{b}={\left[\begin{array}{ccc} \delta l_{x} & \delta l_{y} & \delta l_{z} \end{array}\right]}^{T}$$

As could be seen from Figure 1, when the land vehicle had an angular motion, the relationship of velocities measured by two odometers and SINS could be written as follows:

(2) $$V_{SINS}=V_{D}-\omega_{nb}^{b}\times \delta l^{b}$$

The second item on the right side of the above formula is the velocity difference of the odometer and SINS caused by the lever arm effect.

(3) $$\omega_{nb}^{b}=\omega_{ib}^{b}-\omega_{in}^{b}=\omega_{ib}^{b}-C_{n}^{b}\omega_{in}^{n}=\omega_{ib}^{b}-C_{n}^{b}(\omega_{ie}^{n}+\omega_{en}^{n})$$

where $\omega_{ib}^{b}$ can be directly measured by the gyros of SINS. $C_{n}^{b}$ is the transformation matrix, which can be output in real-time through SINS. $\omega_{ie}^{n}$ is the projection of the rotation angular rate of the earth in the n-frame, and $\omega_{en}^{n}$ is the projection of the angular rate of n-frame related to e-frame in n-frame. $\omega_{ie}^{n}$ and $\omega_{en}^{n}$ can be calculated from the position and velocity information obtained by SINS at the current moment.

### 2.2. Principle of the Integrated Navigation Method

In the proposed integrated method, three FOGs were used to measure the pitch angular rate, roll angular rate, and yaw angular rate of the vehicle, respectively, and three accelerometers were used to measure the forward acceleration, lateral acceleration, and vertical acceleration of the vehicle, respectively. The measurement information was complete and could be used for a navigation solution with the standard strap-down inertial navigation algorithm. After navigation calculation, the system obtained basic navigation information of that time, such as position, velocity, and attitude. At the same time, two different odometers’ velocities, containing lever arm information, were used to carry out the dead-reckoning calculation, as well as to calculate the azimuth.

**Figure 2:**
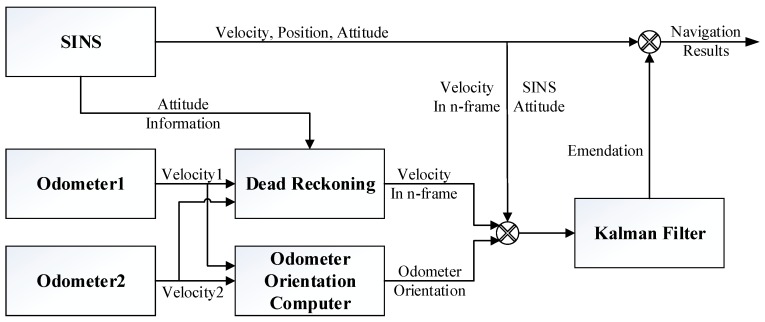
Schematic for the integrated navigation method.

The differences of the velocity and azimuth information between the SINS and couple odometers were taken as the observation data to estimate the state vector of Kalman filter (KF). The error estimations acquired from the Kalman filter would be used to compensate for basic navigation results, including attitude, velocity, and position error estimations. The schematic of the proposed integrated method is shown in Figure 2.

## 3. Error Model Establishment of the Integrated Navigation System

### 3.1. Odometer Velocity Error Model with Lever Arm Effect

There are two matching models for the integration of SINS and odometer. One is the velocity-matching model, and the other is the position-matching model [16,17]. With the proposed method, the odometer was adopted in the velocity matching model to measure the reference velocity of land vehicle moving. However, the real output of the odometer is a series of pulses corresponding to the mileage that the vehicle traveled. While the odometer scale factor *K_D_* was given, the odometer pulse number *N* could deduce the mileage increment *S;* furthermore, the average speed could be calculated with the mileage increment *S* and the time interval Δ*T*. The real output model of the odometer could be written as follows:

(4) $$S=N\cdot K_{D}+W$$

where *N* denotes the pulse number of the odometer in Δ*T*, and *S* represents the mileage increment during the time interval. The odometer ideal scale factor was denoted by *K_D_*, which was calculated by the wheel perimeter, and the odometer white noise was denoted by *W_D_*.

The velocity of the land vehicle in the b-frame $V_{D}^{b}$ could be expressed as:

(5) $$V_{D}^{b}={\left[\begin{array}{ccc} 0 & S/\Delta T & 0 \end{array}\right]}^{T}$$

For the purpose of carrying on the integrated navigation, the velocity in the b frame of the land vehicle needed to be converted into n frame with the attitude of the vehicle. As a result, combined with the transition matrix ${\tilde{C}}_{b}^{n}$ computed by SINS, the velocity of the land vehicle in n-frame $V_{D}^{n}$ could be expressed as:

(6) $$V_{D}^{n}={\tilde{C}}_{b}^{n}\cdot V_{D}^{b}$$

Then, considering (4), (6) could be expressed as:

(7) $$V_{D}^{n}={\tilde{C}}_{b}^{n}{\left[\begin{array}{ccc} 0 & S/\Delta T & 0 \end{array}\right]}^{T}$$

where $V_{D}^{n}={\left[\begin{array}{ccc} v_{DE} & v_{DN} & v_{DU} \end{array}\right]}^{T}$ and $v_{DE},v_{DN},v_{DU}$ represent the velocities of the land vehicle in the n-frame.

Considering the scale factor error $\delta K_{D}$ of odometer, the measured velocity of land vehicle ${\tilde{V}}_{D}^{b}$ originated in the true velocity $V_{D}^{b}$, which could be expressed as:

(8) $${\tilde{V}}_{D}^{b}=(1+\delta K_{D})\cdot V_{D}^{b}$$

Due to the misalignment angle error φ and the lever arm effect, the measured velocity in n-frame of the land vehicle could be expressed as:

(9) $${\tilde{V}}_{D}^{n}={\tilde{C}}_{b}^{n}\cdot {\tilde{V}}_{D}^{b}=(I-\varphi \times )\cdot C_{b}^{n}\cdot ({\tilde{V}}_{D}^{b}-\omega_{nb}^{b}\times \delta l^{b})$$

Ignoring the second-order quantities in the expansion, the right side of (9) could be expressed as:

(10) $${\tilde{V}}_{D}^{n}=V_{D}^{n}-(\varphi \times )\cdot C_{b}^{n}\cdot V_{D}^{b}+C_{b}^{n}\cdot \delta K_{D}\cdot V_{D}^{b}+C_{b}^{n}\cdot (\omega_{nb}^{b}\times )\cdot \delta l^{b}$$

Thus, the velocity error of the odometer $\delta V_{D}^{n}$ could be expressed as:

(11) $$\delta V_{D}^{n}=-(\varphi \times )\cdot C_{b}^{n}\cdot V_{D}^{b}+C_{b}^{n}\cdot \delta K_{D}\cdot V_{D}^{b}+C_{b}^{n}\cdot (\omega_{nb}^{b}\times )\cdot \delta l^{b}$$

where $\delta V_{D}^{n}={\left[\begin{array}{ccc} \delta v_{DE} & \delta v_{DN} & \delta v_{DU} \end{array}\right]}^{T}$ and $\delta v_{DE},\delta v_{DN},\delta v_{DU}$ are the velocity errors of the land vehicle in the n-frame.

### 3.2. Odometer Azimuth Error Model

**Figure 3:**
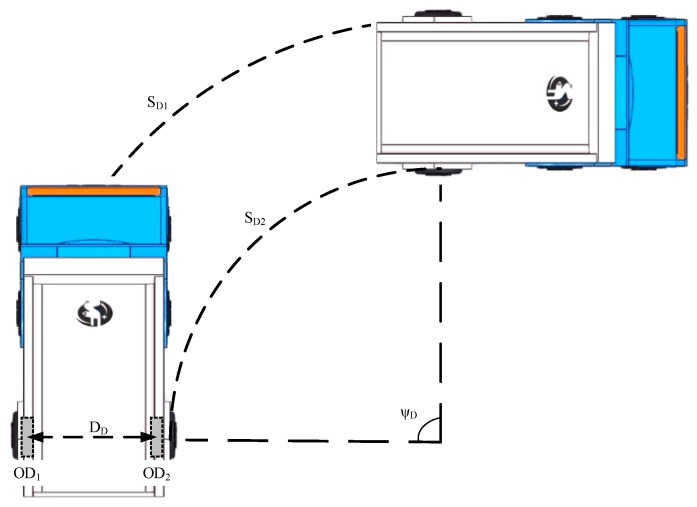
The azimuth of couple odometers.

In the proposed method, a couple of odometers were applied to measuring the reference azimuth of a land vehicle turning on the ground. When the land vehicle turned in traveling, two rear wheels would move forward different distances, and the couple odometers, which were mounted on the rare wheels, would output two different mileages increment *S_D_*_1_ and *S_D_*_2_ separately, as shown in Figure 3. The distance of two rare wheels *D_D_* had been precisely measured before. Then, the azimuth model of the odometer could be written as the following equation:

(12) $$\psi_{D}=\frac{S_{D1}^{b}-S_{D2}^{b}}{D_{D}^{b}}=\frac{(S_{D1}^{b}-S_{D2}^{b})\times \frac{1}{dt}}{(D_{D}^{b})\times \frac{1}{dt}}=\frac{(V_{D1}^{b}-V_{D2}^{b})\times dt}{D_{D}^{b}}$$

where *S_D_*_1_ and *S_D_*_2_ are the mileage increments of the land vehicle during the sampling time *dt*. Use $D_{D}$ to represent the distance of two rear wheels.

Considering the actual velocity measured by the odometer ${\tilde{V}}_{D}^{b}$, the actual azimuth measured by odometer ${\tilde{\psi }}_{D}$ could be written as follows:

(13) $$\begin{array}{cc} {\tilde{\psi }}_{D} & =\psi_{D}+\delta \psi_{D}=\frac{({\tilde{V}}_{D1}^{b}-{\tilde{V}}_{D2}^{b})\times dt}{D_{D}^{b}}=\frac{((V_{D1}^{b}+\delta V_{D1}^{b})-(V_{D2}^{b}+\delta V_{D2}^{b}))\times dt}{D_{D}^{b}}\\ & =\frac{((V_{D1}^{b}-V_{D2}^{b})+(\delta V_{D1}^{b}-\delta V_{D2}^{b}))\times dt}{D_{D}^{b}}=\frac{(V_{D1}^{b}-V_{D2}^{b})\times dt}{D_{D}^{b}}+\frac{(\delta V_{D1}^{b}-\delta V_{D2}^{b})\times dt}{D_{D}^{b}} \end{array}$$

Thus, the azimuth error $\delta \psi_{D}$ could be written as follows:

(14) $$\delta \psi_{D}=\frac{(\delta V_{D1}^{b}-\delta V_{D2}^{b})\times dt}{D_{D}^{b}}=\frac{\delta K(V_{D1}^{b}-V_{D2}^{b})\times dt}{D_{D}^{b}}=\delta K\cdot \psi_{D}$$

### 3.3. SINS Error Model

The SINS employs accelerometers and gyros to measure the acceleration and angular rate of a land vehicle and continuously provides the position, attitude, and velocity information of the vehicle with the principle of strap-down inertial navigation. Nevertheless, there are serval unavoidable errors in SINS, such as inertial sensor errors, structure errors, installation errors, various disturbances, and initial condition of the system. The navigation accuracy of the SINS would be depressed by these unavoidable errors.

The navigation error propagation equations of SINS have been studied many times [18,19,20,21]. So, the error equations in n-frame of the SINS could be expressed as (15):

(15) $$\begin{array}{cc} \dot{\varphi } & =\varphi \times (\omega_{ie}^{n}+\omega_{en}^{n})+\delta \omega_{ie}^{n}+\delta \omega_{en}^{n}-C_{b}^{n}\varepsilon^{b}\\ \delta {\dot{v}}^{n} & =-\varphi \times f^{n}+\delta v^{n}\times (2\omega_{ie}^{n}+\omega_{en}^{n})+v^{n}\times (2\delta \omega_{ie}^{n}+\delta \omega_{en}^{n})+C_{b}^{n}\nabla^{b}\\ \delta \dot{L} & =-\frac{v_{N}}{{\left(R_{M}+h\right)}^{2}}\delta h+\frac{1}{R_{M}+h}\delta v_{N}\\ \delta \dot{\lambda } & =\frac{v_{E}\tan L\cdot \sec L}{R_{N}+h}\cdot \delta L-\frac{v_{E}\sec L}{{\left(R_{N}+h\right)}^{2}}\cdot \delta h+\frac{\sec L}{R_{N}+h}\cdot \delta v_{E}\\ \delta \dot{h} & =\delta v_{U} \end{array}$$

(16) $$\begin{array}{cc} C_{b}^{n} & =\left[\begin{array}{ccc} \cos\gamma \cos\psi +\sin\gamma \sin\psi \sin\theta & \sin\psi \cos\theta & \sin\gamma \cos\psi -\cos\gamma \sin\psi \sin\theta \\ -\cos\gamma \sin\psi +\sin\gamma \cos\psi \sin\theta & \cos\psi \cos\theta & -\sin\gamma \sin\psi -\cos\gamma \cos\psi \sin\theta \\ -\sin\gamma \cos\theta & \sin\theta & \cos\gamma \cos\theta \end{array}\right]\\ & =\left[\begin{array}{ccc} a_{11} & a_{12} & a_{13}\\ a_{21} & a_{22} & a_{23}\\ a_{31} & a_{32} & a_{33} \end{array}\right] \end{array}$$

where *a_ij_* (*i/j* = 1, 2, 3) represents the element in the transformation matrix $C_{b}^{n}$ separately. In n-frame, $\delta v^{n}$ is the velocity error vector. φ is the misalignment angle vector, and δλ, δL, δh, respectively, denote the longitude error, latitude error, and height error. $\nabla^{b}$ and $\varepsilon^{b}$, respectively, denote accelerometer biases and gyro drifts in b-frame. $R_{M}$ and $R_{N}$ separately represent the meridian and the prime vertical radius of the earth.

## 4. High Accuracy Integrated Navigation Algorithm

### 4.1. State Equation of the System

Based on the error equations of SINS and odometer, SINS errors and two odometers’ errors, including scale factor error and lever arm errors, were selected as state variables. The state equation of the proposed integrated method could be established as:

(17) $$\dot{X}(t)=FX(t)+GW(t)$$

where *F* is the system transformation matrix, which could be obtained according to (15). *G* represents the transformation matrix of the system noise. Use *W*(*t*) to represent the white noise of the system. The 22-dimension state vector of the integrated system *X*(*t*) could be expressed as:

(18) $$\begin{array}{c} X(t)=[\begin{array}{cccc} \phi_{E} & \phi_{N} & \phi_{U} & \begin{array}{cccc} \delta v_{E} & \delta v_{N} & \delta v_{U} & \begin{array}{ccc} \delta L & \delta \lambda & \begin{array}{cccc} \delta h & \varepsilon_{x} & \varepsilon_{y} & \varepsilon_{z} \end{array} \end{array} \end{array} \end{array}\\ \begin{array}{cccc} \nabla_{x} & \nabla_{y} & \nabla_{z} & \begin{array}{cccc} \begin{array}{cccc} \delta K_{D} & \delta l_{x1} & \delta l_{y1} & \delta l_{z1} \end{array} & \delta l_{x2} & \delta l_{y2} & \delta l_{z2}{]}^{T} \end{array} \end{array} \end{array}$$

(19) $$W(t)={\left[\begin{array}{ccc} w_{gx} & w_{gy} & \begin{array}{ccc} w_{gz} & w_{ax} & \begin{array}{ccc} w_{ay} & w_{az} & 0_{1\times 16} \end{array} \end{array} \end{array}\right]}^{T}$$

when the vehicle is driving normally on the ground, the inertial sensor errors, such as accelerometer biases ∇ and FOG drifts ε, could be reduced to the sum of random noise and deviation. Biases and drifts could be regarded as the constant in a short time:

(20) $$\left\{ \begin{array}{c} \dot{\varepsilon }=0\\ \dot{\nabla }=0 \end{array}\right.$$

Besides the SINS errors, the scale factor error of the odometer is another important error of the integrated system. The outputs of odometers are usually affected by systemic or non-systematic factors, such as resolution, limited sampling rate, the wheels’ sliding or idling, passing through objects on the road, tire inflation [22]. Before use, the scale factor of the odometer is usually calibrated, and the value of calibration would not change in a short time. Consequently, during working hours, the scale factor error could be considered as a constant:

(21) $$\delta {\dot{K}}_{D}=0$$

Similarly, in the practical situation, the SINS and the couple odometers are firmly installed on the land vehicle, and the relative lever arm parameters of the SINS and odometers would not change during the land vehicle traveling. Therefore, the lever arm parameters could be defined as constants during the working time:

(22) $$\delta \dot{\vec{l}}=0$$

### 4.2. Measurement Equation of the System

In the integrated navigation system, the couple odometers provide two different velocity information and azimuth information of the land vehicle. The differences between the output acquired from the SINS and the couple odometers are chosen as an observation vector of the integrated navigation system:

(23) $$Z(t)=\left[\begin{array}{c} v_{SINS}-v_{D1}\\ v_{SINS}-v_{D2}\\ \psi_{SINS}-\psi_{D} \end{array}\right]$$

which could be expressed as:

(24) $$Z(t)={\left[\begin{array}{cccc} v_{E}-v_{D1E} & v_{N}-v_{D1N} & v_{U}-v_{D1U} & \begin{array}{cccc} v_{E}-v_{D2E} & v_{N}-v_{D2N} & v_{U}-v_{D2U} & \psi_{SINS}-\psi_{OD} \end{array} \end{array}\right]}^{T}$$

Taking errors that the SINS and odometers have into consideration, (24) could be expressed as:

(25) $$Z(t)=\left[\begin{array}{c} \begin{array}{c} \delta v_{E}-\delta v_{D1E}\\ \delta v_{N}-\delta v_{D1N}\\ \delta v_{U}-\delta v_{D1U}\\ \delta v_{E}-\delta v_{D2E} \end{array}\\ \delta v_{N}-\delta v_{D2N}\\ \delta v_{U}-\delta v_{D2U}\\ \delta \psi_{SINS}-\delta \psi_{OD} \end{array}\right]$$

where substituting (11) and (14) into (25), and (25) could be extended as follows:

(26) $$Z(t)=\left[\begin{array}{c} \delta v_{E}+v_{U}\phi_{N}-v_{N}\phi_{U}-v_{E}\delta K_{D}+(a_{12}\omega_{z}-a_{13}\omega_{y})l_{x1}+(-a_{11}\omega_{z}+a_{13}\omega_{x})l_{y1}+(a_{11}\omega_{y}-a_{12}\omega_{x})l_{z1}\\ \delta v_{N}-v_{U}\phi_{E}+v_{E}\phi_{U}-v_{N}\delta K_{D}+(a_{22}\omega_{z}-a_{23}\omega_{y})l_{x1}+(-a_{21}\omega_{z}+a_{23}\omega_{x})l_{y1}+(a_{21}\omega_{y}-a_{22}\omega_{x})l_{z1}\\ \begin{array}{l} \delta v_{U}+v_{N}\phi_{E}-v_{E}\phi_{N}-v_{U}\delta K_{D}+(a_{32}\omega_{z}-a_{33}\omega_{y})l_{x1}+(-a_{31}\omega_{z}+a_{33}\omega_{x})l_{y1}+(a_{31}\omega_{y}-a_{32}\omega_{x})l_{z1}\\ \delta v_{E}+v_{U}\phi_{N}-v_{N}\phi_{U}-v_{E}\delta K_{D}+(a_{12}\omega_{z}-a_{13}\omega_{y})l_{x2}+(-a_{11}\omega_{z}+a_{13}\omega_{x})l_{y2}+(a_{11}\omega_{y}-a_{12}\omega_{x})l_{z2}\\ \delta v_{N}-v_{U}\phi_{E}+v_{E}\phi_{U}-v_{N}\delta K_{D}+(a_{22}\omega_{z}-a_{23}\omega_{y})l_{x2}+(-a_{21}\omega_{z}+a_{23}\omega_{x})l_{y2}+(a_{21}\omega_{y}-a_{22}\omega_{x})l_{z2}\\ \delta v_{U}+v_{N}\phi_{E}-v_{E}\phi_{N}-v_{U}\delta K_{D}+(a_{32}\omega_{z}-a_{33}\omega_{y})l_{x2}+(-a_{31}\omega_{z}+a_{33}\omega_{x})l_{y2}+(a_{31}\omega_{y}-a_{32}\omega_{x})l_{z2} \end{array}\\ -\phi_{U}-\delta K\cdot \psi_{od} \end{array}\right]$$

As a result, the measurement equation of the integrated system could be described by:

(27) Z(t)=HX(t)+V(t)

where *H* represents the measurement matrix. *Z*(*t*) is the measurement of the system, and *V*(*t*) is the Gaussian white noise sequence. The matrix *H* could be presented by the following equation:

(28) $$H=\left[\begin{array}{cccc} \begin{array}{l} 0\\ -v_{DU}\\ v_{DN}\\ 0 \end{array} & \begin{array}{l} v_{DU}\\ 0\\ -v_{DE}\\ 0 \end{array} & \begin{array}{l} -v_{DN}\\ v_{DE}\\ 0\\ -1 \end{array} & \begin{array}{cccc} \begin{array}{l} 1\\ 0\\ 0\\ 0 \end{array} & \begin{array}{l} 0\\ 1\\ 0\\ 0 \end{array} & \begin{array}{l} 0\\ 0\\ 1\\ 0 \end{array} & \begin{array}{cc} 0_{3\times 9} & \begin{array}{l} -v_{DE}\\ \begin{array}{cc} -v_{DN} & C_{b}^{n}\cdot (\omega_{nb}^{b}\times ) \end{array}\\ -v_{DU}\\ \begin{array}{cc} -\psi_{OD} & 0_{1\times 3} \end{array} \end{array} \end{array} \end{array} \end{array}\right]$$

## 5. Simulation and Result Analysis

In order to verify the effectiveness of the proposed integrated method, two integrated navigation methods would be implemented for comparison: one was the proposed integrated navigation system of couple odometers and FOG SINS presented in Section 2, and the other was the traditional integrated navigation system of single odometer and FOG SINS.

Simulations were carried out with MATLAB software, and all parameters used in simulations coincided with the actual SINS and couple odometers integrated system, based on FOGs and accelerometers, which are depicted in Table 1. In addition, the initial positions of the two systems were 116.34682° E, 39.97885° N, and the operating frequency of the integrated navigation system was 100 Hz.

The initial alignment results of two integrated navigation methods were exactly alike since the same FOG SINS and the same initial alignment algorithm were applied in two integrated systems. In FOG SINS, the initial alignment errors were mainly caused by constant inertial sensor errors, such as gyroscope drifts and accelerometer biases. According to the device precision of the inertial sensors in Table 1, the initial misalignment results of two integrated navigation systems could be set, as shown in Table 2.

**Figure 4:**
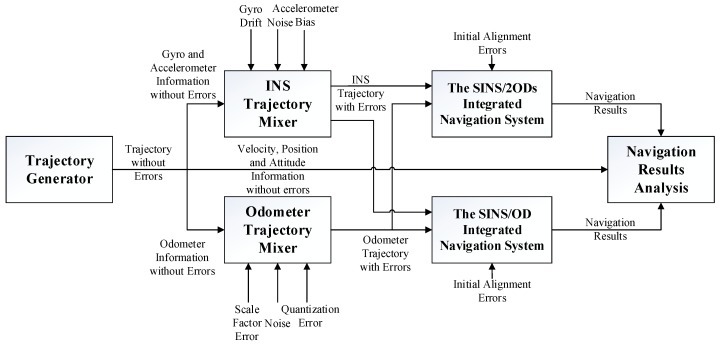
The procedure of the simulation.

Applying the parameters described above, two integrated navigation systems were implanted with the trajectory, as described in Table 3. Figure 4 illustrates the procedure of simulations. The maximum speed of the vehicle was 20 m/s, and the entire trajectory was approximately 95 km.

The percentage of the horizontal position error *δP* was usually adopted to represent the precision of the horizontal position of the land vehicle, which was calculated using the following formula:

(29) $$\delta P=0.5887\times \left(\delta P_{N}+\delta P_{E}\right)/D$$

where $\delta P_{N}$ and $\delta P_{E}$, respectively, represent horizontal position errors in the direction of north and east. *D* is the current mileage of the land vehicle.

**Figure 5:**
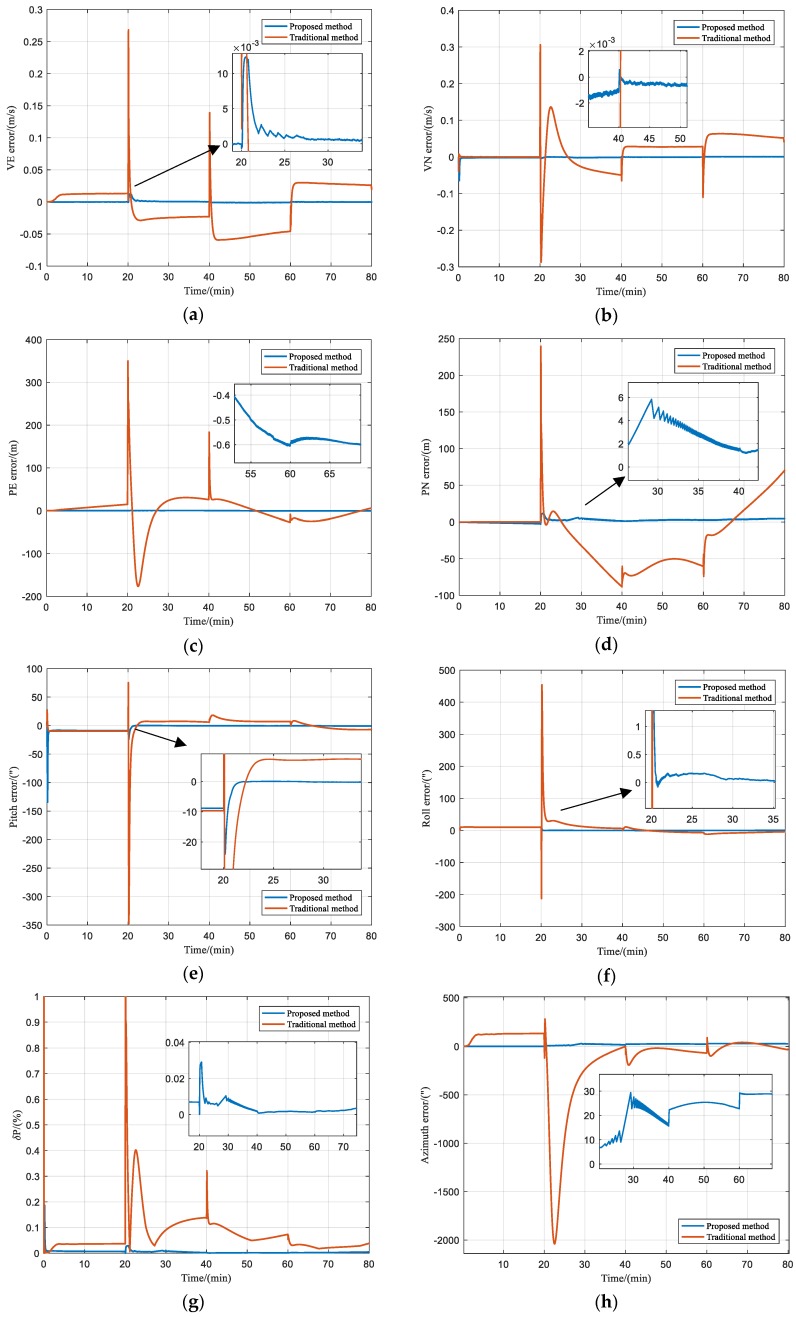
(**a**) *V_E_* errors; (**b**) *V_N_* errors; (**c**) *P_E_* errors; (**d**) *P_N_* errors; (**e**) Pitch errors; (**f**) Roll errors; (**g**) The percentage of horizontal position errors; (**h**) Azimuth errors.

For simplicity, only a few typical results are shown in Figure 5 to describe attitude errors, position errors, and velocity errors, rather than all simulation results.

It could be known from Figure 5a–d that the *P_E_* error of the proposed integrated method was no more than 1 m, and the *V_E_* error was within 0.02 m/s throughout the entire trajectory. But in the traditional method, the *V_E_* error was divergent with time to the maximum of 0.45 m/s, and the *P_E_* error was about ±40 m. Besides, when the land vehicle turned, both *V_E_* and *P_E_* of the traditional method had obvious errors. The reason was that without considering the lever arm effect, the traditional integrated method could not estimate and compensate for the errors with two inconsistent velocity measurement information. In addition, as could be seen from Figure 5e,f, both roll and pitch errors of the proposed integrated method had good convergence and did not exceed ±2″. Nevertheless, the traditional integrated method had pitch and roll errors of about ±10″. During the first turn, the horizontal attitudes had larger errors because of the lever arm effect, and both attitude errors converged after the first turn.

Comparisons of the azimuth error and the horizontal position error of two integrated navigation methods are shown in Figure 5g,h, respectively. The results demonstrated that before the first turn, the maximum *δP* of the traditional method was 0.05% D (the horizontal position error was about 14 m), and the azimuth error was approximately 140″. During the first turn, both *δP* and azimuth error of the traditional method had obvious errors because of the lever arm effect. Then, the average *δP* and the azimuth error gradually decreased to 0.03% D (the horizontal position error was about 30 m) and ±100″ after the first turn. The reason was that the observability of the integrated system became better, and navigation errors were effectively estimated and compensated with the integrated navigation algorithm. The maximum *δP* of the proposed integrated navigation method was 0.03% D (the horizontal position error was about 7 m) before the first turn, as well as the azimuth error quickly converged to less than ±30″. After the first turn, the average *δP* further decreased to 0.01% D (the horizontal position error was about 11 m). Moreover, the accuracy of the horizontal position had further improvement after the second and the third turns. Consequently, compared with the traditional integrated method, the positioning and orientation accuracy of the proposed integrated navigation method was greatly improved.

## 6. Conclusions

This paper proposed a new integrated navigation method based on couple odometers and FOG SINS. Couple odometers were used to measure the velocity and azimuth of a land vehicle. The error model of the SINS/2ODs integrated system was analyzed, and the dead-reckoning algorithm compensating the lever arm effect of two odometers was proposed. At the same time, the velocity differences of two odometers were used to calculate the vehicle’s azimuth, which was taken as azimuth measurement information of the integrated navigation system. Then, several long-term navigation simulations were implemented to verify the effectiveness of the proposed integrated navigation method, and the results indicated that the proposed method could be used in different scenarios, and the positioning and orientation accuracy of the proposed method could be markedly improved as compared with the traditional method. Therefore, the proposed integrated navigation method based on FOG SINS and couple odometers could effectively improve not only positioning accuracy but also orientation accuracy of the land vehicle, thus having greater application value.

## Figures and Tables

**Table 1:** Parameters of the system devices.

**Devices**	**Drift or Bias**	**Scale Factor Error**	**Stochastic Noise**
Gyroscope X	0.01°/h	20 ppm	0.01°$/\sqrt{h}$
Gyroscope Y	0.01°/h	20 ppm	0.01°$/\sqrt{h}$
Gyroscope Z	0.01°/h	20 ppm	0.01°$/\sqrt{h}$
Accelerometer X	50 µg	20 ppm	5 × 10^−5^ $g\cdot \sqrt{s}$
Accelerometer Y	50 µg	20 ppm	5 × 10^−5^ $g\cdot \sqrt{s}$
Accelerometer Z	50 µg	20 ppm	5 × 10^−5^ $g\cdot \sqrt{s}$
	**Quantization Error**	**Scale Factor Error**	**Stochastic Noise**
Odometer 1	1 pulse	0.1%	0.1%
Odometer 2	1 pulse	0.1%	0.1%

**Table 2:** Initial misalignment angles.

Method	Misalignment	Value/(″)
SINS/2ODs integrated navigation	$\varphi_{E}$	10
	$\varphi_{N}$	10
	$\varphi_{U}$	180
SINS/OD integrated navigation	$\varphi_{E}$	10
	$\varphi_{N}$	10
	$\varphi_{U}$	180

**Table 3:** The trajectory of the land vehicle.

Procedure	Acceleration or Angular Rate	Time
(1) Static (the initial heading of the vehicle is north)	0	0s
(2) Accelerate	2 m/s^2^	0 s–10 s
(3) Uniform motion	0	10 s–1200 s
(4) Turn right	18°/s	1200 s–1205 s
(5) Uniform motion	0	1205 s–2400 s
(6) Turn right	18°/s	2400 s–2405 s
(7) Uniform motion	0	2405 s–3600 s
(8) Turn right	18°/s	3600 s–3605 s
(9) Uniform motion	0	3605 s–4790 s
(10) Decelerate	–2 m/s^2^	4790 s–4800 s
